# Supplementary material for: Entropy-Based Temporal Downscaling of Precipitation as Tool for Sediment Delivery Ratio Assessment
Source: Entropy (Basel). 2021 Dec 1;23(12):1615. doi: 10.3390/e23121615 (PMC8700339; doi:10.3390/e23121615)
Supplement: Supplementary file 1 [file entropy-23-01615-s001.zip › entropy-1425890-Supplementary.pdf]

Supplements to:

Entropy-based temporal downscaling of precipitation as tool for sediment delivery ratio assessment

Pedro Henrique Lima Alencar[1,2], Eva Nora Paton[1], José Carlos de Araújo[2]

[1]Ecohydrology and Landscape Evaluation, Institute of Ecology, Technische Universität Berlin, Germany

[2] Ecohydrology and Landscape Evaluation, Institute of Ecology, Technische Universität Berlin, Germany

Corresponding Author: Pedro Alencar, Ecohydrology and Landscape Evaluation, Institute of Ecology, TU Berlin, ErnstReuterPlatz 1, Room 808, 10587 Berlin, Germany. Email: pedro.alencar@campus.tuberlin.de

1 INTRODUCTION

In Figure S1 we present the map of pluviometric stations with daily and sub-daily data in the Brazilian Northeastern Region. As discussed in the main text, there are few stations monitoring sub-daily precipitations in the region. Additionally, most stations in the left of the plot have a short time series and/or with gaps.

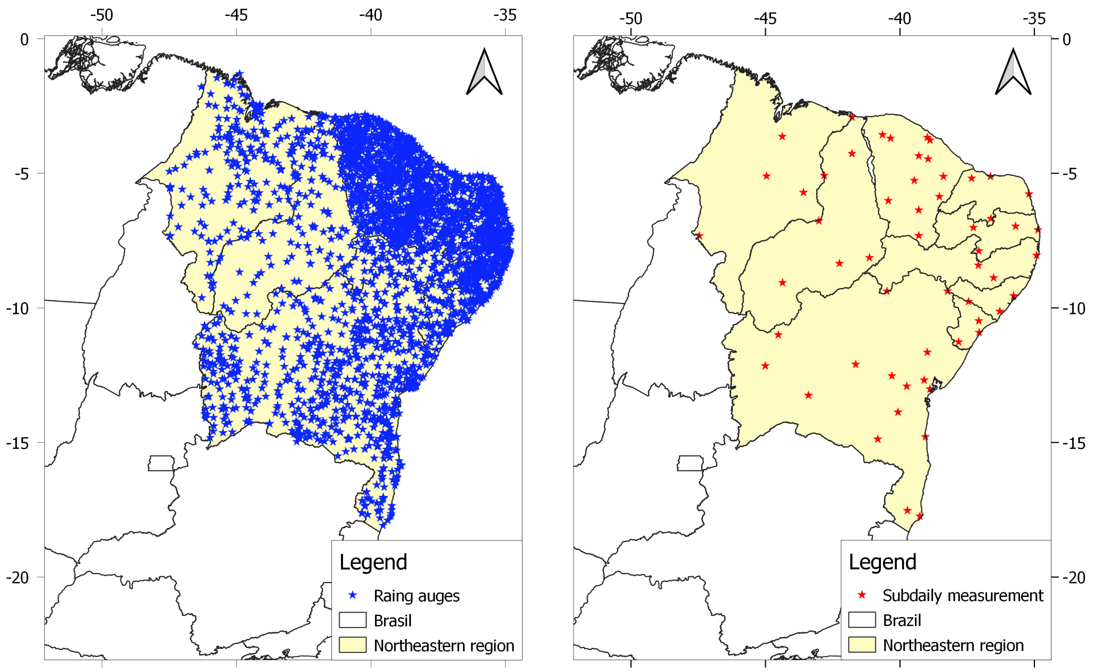

Figure S1: Map of Ville de Paris and Automatic stations in the Brazilian Semiarid Managed by the Brazilian National Water Agency (ANA, 2019).

2 MATERIALS AND METHODS

2.1 The Principle of Maximum Entropy

The Principle of Maximum Entropy (PoME) is grounded on the concept of entropy as a measure of uncertainty or information, as proposed by (Shannon, 1948). Based on abstraction, Jaynes (1957a,b) proposed the PoME to obtain the least-biased probability function on the basis of known information represented as constrains. The Shannon entropy equation is expressed as (Eq. S1):

$$h_x = -\int f(x) \ln f(x) dx \tag{S1}$$

h_x is the total entropy for the variable x. The function $f(x)$ that maximises h_x is the one that does not consider any non-proved hypothesis. To maximize Eq. S1, subjected to the constrains, we can formulate the Lagrangian function $\mathcal{L}$ (Eq. S2) and differentiate in respect to f and equals the derivative to zero (Eq. S3).

$$\mathcal{L} = -\int_{x_0}^{x_1} f(x) \ln f(x) dx - \sum_{r=0}^{n} \lambda_r \left[\int_{x_0}^{x_1} f(x)\, g_r(x) dx - C_r \right] \tag{S2}$$

$$\frac{\partial \mathcal{L}}{\partial f} = 0 \rightarrow \frac{\partial \mathcal{L}}{\partial f} = -1 - \ln f(x) - \sum_{r=0}^{n} \lambda_r\, g_r(x) = 0 \tag{S3}$$

$\lambda_0, \lambda_1, \ldots, \lambda_n$ are the Lagrange multipliers. $g_r(x)$ are functions of x related to the constraints. n is the number of restrictions besides the trivial ($r = 0 \longrightarrow \int f(x) dx = 1$). Solving Equation S3 for $f(x)$ one finds the probability distribution in terms of the Lagrange multipliers as in Eq S4.

$$f(x) = \exp\left[-\sum_{r=0}^{n} \lambda_r\, g_r(x) \right] \tag{S4}$$

2.2 The SYPoME Model

Proposed by de Araújo (2007), the SYPoME (Sediment Yield Model based on the Principle of Maximum Entropy) allows the user to assess the hillslope sediment production of each event and is given by Equation S5:

$$Q_s = \bar{\varepsilon}\, A\, SDR = \bar{\varepsilon}\, A \frac{e^{\lambda L_m} (L_0 - x_0)\, \lambda - \left(e^{\lambda (L_0 - x_0)} - 1 \right)}{\lambda L_0 \left(e^{\lambda (x_0 + L_m)} - 1 \right)} \tag{S5}$$

$\bar{\varepsilon}$ (Mg ha^{-1} yr^{-1}) is the gross erosion obtained, for example, by using the Universal Soil Loss Equation (USLE Wischmeier and Smith, 1978), A (ha) the hillslope contribution area, L_0 the hill slope length (m), L_m the maximum sediment travel distance (m), x_0 is the initial position of erosion in the hillslope and λ is a Lagrange multiplier. The ratio of the sediment portion

that reaches rivers and promotes siltation (Q_s) and all mobilised sediment ($\bar{\varepsilon}\,A$). The *SDR* is restricted to a closed interval (*SDR* $\in [0,1]$).

The parameters λ and L_m can be obtained by solving the systems of equations derived with the PoME (Eq. S6)

$$\begin{cases} \frac{1}{L_m} = \frac{e^{\lambda\,(x_0+L_0)/2}}{e^{\lambda\,(x_0+L_m)}-1} \\ \frac{e^{\lambda\,(x_0+L_m)}\,[\lambda\,(L_m+x_0)-1]-e^{\lambda\,x_0}\,(\lambda\,x_0-1)}{\lambda\left(e^{\lambda\,(x_0+L_m)}-1\right)} = K_v\left(\frac{\rho_s}{\rho_s-\rho}\right)\frac{\Omega\,L_0}{g\,\bar{\varepsilon}\,v_s} \end{cases} \tag{S6}$$

g (m s^{-2}) is the gravity, ρ (kg m^{-3}) is the density of water, ρ_s (kg m^{-3}) is sediment density, Ω (J s^{-1} m^{-2}) the stream power (Eq. S7) according to Bagnold (1977), v_s (m s^{-1}) is the sediment settling velocity and K_v is the delivery parameter related to surface conditions, which be calibrated or obtained as function of the parameters CP of the USLE. The system of Equations S6 allows us to obtain the two parameters necessary to calculate the *SDR*.

$$\Omega = \rho\,g\,S_0\,R_H\,U \tag{S7}$$

S_0 (m m^{-1}) is the slope; R_H (m) the hydraulic radius that can be approximated to the flow depth for wide hills; and U (m s^{-1}) is the flow velocity. In his original work, de Araújo (2007) achieved good results (average absolute error 20%) with the model by using the average velocity for each event, given by Equation S8.

$$\bar{U} = \left(\frac{D}{H_e}\right)^{-1} \tag{S8}$$

H_e (mm) is the effective precipitation or total runoff and D (s) the total duration of the event. Hence, instead of requiring the knowledge of the complete hydrograph, we only need the information on the effective precipitation initiation and on its end, usually unavailable.

2.3 Gross Erosion Assessment

The Universal Soil Loss Equation (Wischmeier and Smith, 1978) is an empirical equation with simple implementation as expressed by the product below (Eq. S9):

$$\bar{\varepsilon} = R\,K\,LS\,C\,P \tag{S9}$$

R (rainfall and runoff factor or erosivity factor) represents the total energy of an event or a series of events which may produce erosion; K (erodibility factor) indicates how much the soil in the studied area is prone to be mobilised by the rain energy; LS (topographic factor) is the length factor and S the slope factor, directly connected to the topography; C (cover and management factor) is a measure of the effect of all cover and management variables, such as type and condition of vegetation and tillage practices; and P (management practice factor) accounts for good practices to reduce erosion, as contouring and terracing.

2.3.1 Erosivity Factor (R)

In order to calculate the gross erosion by employing the USLE we need to assess the erosivity value (R MJ ha^{-1} h^{-1}). We used two approaches:

i. Probabilistic approach

Based on measured data concerning sub-daily precipitation, we studied the best probabilistic distribution (uniform, gaussian, two-parameter gamma and beta distributions were tested) for the variable I_{30}/H. Using an estimated I_{30} (mm h^{-1}) we calculated the event erodibility using Equation S10

$$R = E\,I_{30} \tag{S10}$$

$$E = \begin{cases} 11.9 + 8.73\log_{10}\bar{I} & \forall\, H < 76.2mm \\ 28.3 & \forall\, H \geq 76.2mm \end{cases} \tag{S11}$$

where E is a storm's kinetic energy, given by the Equation S10. In Eq. S11 above, H (mm) is the total precipitation and $\bar{I}$ (mm h$_{-1}$) the average intensity. Note that we obtain $\bar{I}$ as the ratio H/D.

ii. Regional approach

Using measured data of rainfall intensities in a semiarid region (de Figueiredo et al., 2016), an equation for the monthly erosivity was calibrated. Events erosivity was obtained by distributing the months erosivity proportionally to the events total precipitation within the month (Eq. S12).

$$\begin{cases} R_m = \alpha \left(\frac{H_m^2}{H_a}\right)^{\beta} \\ R_{i,m} = \frac{R_m H_{i,m}}{H_m} \end{cases} \tag{S12}$$

R_m is the months total erosivity, H_m the months total precipitation, H_a the average annual precipitation, and $R_{i,m}$ the erosivity of the $i-th$ event of the month m, and $H_{i,m}$ the precipitation of the $i-th$ event of the month m; α and β are regional calibrated parameters equalling 565 and 0.42 respectively.

2.3.2 Erodibility factor (K)

Soil erodibility was estimated using the Soil Classification Maps of Brazil (IPECE, 2007) and the correspondent erodibility factor as obtained experimentally by Silva (1978).

2.3.3 Topography factor (LS)

The topography factor was calculated applying equation S13.

$$LS = 0.00984\, L_r^{0.63}\, S^{1.18} \tag{S13}$$

$$L_r = \frac{A_q}{4 \sum L_{dem}} \tag{S14}$$

where S is the slope in percentage and L_r is the average slope length, given by equation S14. A_q is the area of the pixel, sub-basin, or landscape unity and $\sum L_{den}$ the sum of all water paths within A_q.

2.3.4 Cover and management practice factor (CP)

We used satellite images (LandSat 8) and field surveys in order to identify the land use. From land use maps the parameter C was mapped using the values of table 8.8 of Haan et al. (1994,

p. 266). The practice factor P was assumed equals the unity, since no management practices where identified in the areas.

2.4 Runoff

To estimate the total runoff per event we used the Soil Conservation Service (1972) Curve Number method (Eq. S15). The *CN* value was estimated on the basis of land use, soil properties and antecedent moisture (Mishra and Singh, 2003). I_a accounts for all initial abstractions and S for the potential maximum retention of the catchment, all in millimetres. I_a is often represented as a fraction ϕ of S. In this study, ϕ was assumed equals 0.20 for all study areas. S is a function of *CN* (Eq. S16).

$$H_e = \frac{(H - I_a)^2}{(H - I_a + S)} \tag{S15}$$

$$S = 25.4\left(\frac{1000}{\text{CN}} - 10\right) \tag{S16}$$

The duration of the runoff was assumed to be equal to the duration of rainfall for the small catchments (< 10 hectares). For the medium, such as Aiuaba and Canabrava, field measurements suggest a duration, on average, 2.5 times longer than the rainfall (de Figueiredo et al., 2016) and for the larger catchments we used the Snyder (1938) Unit Hydrograph.

2.5 USLE Data

In Table S1 we present the values of the USLE parameters obtained accordingly to Wischmeier and Smith (1978). The parameter P was assumed equal to one, for no management practices were identified in the regions.

Table S1: Average characteristics of the study areas - LULC, arae and USLE parameters

Name	Land Use	Area (km^2)	Slope (%)	K[a]	L (-)	S (-)	C (-)
Canabrava	Agriculture and open range cattle rasing	2.9	6.6%	0.032	3.252	0.606	0.01
Aiuaba	Conservation area with native vegetation (Caatinga)	11.53	18.0%	0.015	3.16	1.944	0.0005
Várzea da Volta	Agriculture and open range cattle raising	155	22.1%	0.028	3.766	2.364	0.028
Acarape	Agriculture and open range cattle raising	208	10.1%	0.037	2.766	1.115	0.015
Sumé 2	Experimental area - Preserved vegetation (Caatinga)	0.0107	6.1%	0.021	1.126	0.523	0.008
Sumé 4	Experimental area - Degraded land without vegetation	0.0048	6.8%	0.021	0.848	0.64	1.000
Gilbués	Abandoned land under desertification process without vegetation	0.0004	15.6%	0.007	1.083	1.698	0.771

[a] K in ($Mg\ h\ MJ^{-1}\ mm^{-1}$)

3 Code - SYPoME

```
      PROGRAM SYPoME

!     PROGRAM TO SIMULATE SEDIMENT YIELD USING POME-EQUATION
!      1. VARIABLES DECLARATION

      INTEGER nprec,iprec,ncell,icell,nev,iev,irep,i
      CHARACTER arquivo1*20,arquivo2*20
      CHARACTER*8, DIMENSION(10,3000) :: dia
      INTEGER, DIMENSION(10,3000) :: id
      REAL, DIMENSION(10,3000) :: D,dur,R
      COMMON /EVENTOS/ id,D,dur,R
      INTEGER, DIMENSION(100) :: igauge
      REAL ds,vs,A,K,CP,S0,S,w0,fL,L0,Kv
      COMMON /CELULAS/ ds,vs,A,K,CP,S0,S,w0,fL,L0,Kv,igauge

!      2. MAIN PROGRAM

      CALL ABERTURA(arquivo1,arquivo2,nprec,ncell,nev,irep)

!     read rainfall-related data of the events
      iprec = 0
      DO WHILE (iprec.lt.nprec)
         iprec=iprec+1
         READ(20,*) i
         IF (i.ne.iprec) THEN
            WRITE(*,*)  ' Incompatibility between indexes of gauge stations !!!'
            WRITE(21,*) ' Incompatibility between indexes of gauge stations !!!'
         ENDIF
         WRITE(21,201) ' Precipitation gauge number ..................... ',iprec
         WRITE(21,*)   '-------------------------------------------------------------'
         WRITE(21,*)   '   i     id     date      D(mm)     Dur(min)    R(MJ.mm/ha/h)      '
         WRITE(21,*)   '-------------------------------------------------------------'
         WRITE(*,201)  ' Precipitation gauge number ....1................ ',iprec
         WRITE(*,*)    '-------------------------------------------------------------'
         WRITE(*,*)    '   i     id     date      D(mm)     Dur(min)    R(MJ.mm/ha/h)      '
         WRITE(*,*)    '-------------------------------------------------------------'
         iev = 0
         DO WHILE (iev.lt.nev)
            iev=iev+1
            READ(20,*) id(iprec,iev),dia(iprec,iev),D(iprec,iev),dur(iprec,iev),R(iprec,iev)
            WRITE(21,202) iev,id(iprec,iev),dia(iprec,iev),D(iprec,iev), +
         ENDDO
      WRITE(21,*)     '-------------------------------------------------------------'
      WRITE(*,*)     '-------------------------------------------------------------'
      ENDDO

!     read physiographic-related data of the cells and compute sediment yield
```

```
      SSY = 0
      SGEr = 0
      icell = 0
      DO WHILE (icell.lt.ncell)
         icell = icell+1
         READ (20,*) icell,ds,vs,A,K,CP,S0,w0,Kv,igauge(icell)
         CALL CALCSY(icell,nev,SY,GEr,irep)
         SSY = SSY + SY
         SGEr = SGEr + GEr
      ENDDO

!     close program
      WRITE(21,203) ' Watershed gross erosion (kg) .............',SGEr
      WRITE(21,203) ' Watershed sediment yield (kg) ...........',SSY
      WRITE(21,204) ' Watershed average delivery ratio .........',SSY/SGer
      WRITE(21,*)
      WRITE(21,*)   ' Program concluded successfully.'
      WRITE(*,203) ' Watershed gross erosion (kg) .............',SGEr
      WRITE(*,203) ' Watershed sediment yield (kg) ...........',SSY
      WRITE(*,204) ' Watershed average delivery ratio .........',SSY/SGer
      WRITE(*,*)
      WRITE(*,*)    ' Program concluded successfully.'
      CLOSE(20)
      CLOSE(21)
  201 FORMAT (a50,i4)
  202 FORMAT (i5,2x,i5,2x,a8,2x,f6.2,5x,f8.1,5x,f7.1)
  203 FORMAT (a44,e10.4)
  204 FORMAT (a44,f5.3)
      END

!     3. SUBROUTINE THAT OPENS PROGRAM

      SUBROUTINE ABERTURA(arquivo1,arquivo2,nprec,ncell,nev,irep)

      CHARACTER arquivo1*20,arquivo2*20,title*20
      INTEGER   nprec,ncell,nev,irep

      WRITE(*,*)' SEDIMENT-YIELD ESTIMATION - SYPOME3'
      WRITE(*,*)
      WRITE(*,*)' * Version 3'
      WRITE(*,*)' * SY equation based on the principle of maximum entropy'
      WRITE(*,*)' * Program can only compute up to 3000 events'
      WRITE(*,*)'                         '
      WRITE(*,*)' Universidade Federal do Ceara'
      WRITE(*,*)' Jose Carlos de Araujo'
      WRITE(*,*)' Technische Universitat Berlin'
      WRITE(*,*)' Pedro Alencar'
      WRITE(*,*)' 2019'
      WRITE(*,*)
```

```fortran
      WRITE(*,*) '-----------------------------------------------------------------'

      WRITE(*,*)'Type the name of the input file:'
      READ(*,302) arquivo1
      OPEN(20,file=arquivo1,status='old')
      READ(20,*) title
!       OPEN(20,file='in.txt',status='old')
      WRITE(*,*)
      WRITE(*,*)'Type the name of the output file:'
      READ(*,302) arquivo2
      OPEN(21,file=arquivo2,status='new')
      WRITE(*,*)
!       OPEN(21,file='out.txt',status='new')
      WRITE(*,*)'Do you need a complete (1) or a simplified (2) report?'
      READ(*,*) irep
      IF(irep.ne.1.and.irep.ne.2) THEN
         WRITE(*,*) 'The number is not an option. Default (complete) report will be provided'
         irep = 1
      ENDIF
!      irep = 2

      WRITE(21,*)' SEDIMENT-YIELD ESTIMATION - SYPOME3'
      WRITE(21,*)
      WRITE(21,*)' * Version 3'
      WRITE(21,*)' * SY equation based on the principle of maximum entropy'
      WRITE(21,*)' * Program can only compute up to 3000 events'
      WRITE(21,*)'                          '
      WRITE(21,*)' Universidade Federal do Ceara'
      WRITE(21,*)' Technische Unibversitat Berlin'
      WRITE(21,*)' Jose Carlos de Araujo'
      WRITE(21,*)' Pedro Alencar'
      WRITE(21,*)' 2019'
      WRITE(21,*)
      WRITE(21,*) '-----------------------------------------------------------------'
      WRITE(21,*) 'Title: ', title
      WRITE(*,*) 'Title: ', title
      WRITE(21,*) '-----------------------------------------------------------------'
      WRITE(21,301) ' Input file ..................................... ',"defaut"
      WRITE(21,301) ' Output file .................................... ',"defaut"
      WRITE(*,*) '-----------------------------------------------------------------'
      WRITE(*,301) ' Input file ..................................... ',"defaut"
      WRITE(*,301) ' Output file .................................... ',"defaut"

      READ(20,*) nprec
      READ(20,*) ncell
      READ(20,*) nev
      WRITE(21,303) ' Number of precipitation gauges ................. ',nprec
      WRITE(21,303) ' Number of cells ................................ ',ncell
      WRITE(21,303) ' Number of events ............................... ',nev
```

```fortran
      WRITE(21,*)    '--------------------------------------------------------------'
      WRITE(*,303)   ' Number of precipitation gauges .................  ',nprec
      WRITE(*,303)   ' Number of cells ................................  ',ncell
      WRITE(*,303)   ' Number of events ..............................  ',nev
      WRITE(*,*)     '--------------------------------------------------------------'

  301 FORMAT (a50,a20)
  302 FORMAT (a20)
  303 FORMAT (a50,i4)
      END

!     4. SUBROUTINE THAT COMPUTES SEDIMENT YIELD

      SUBROUTINE CALCSY(icell,nev,SY,GEr,irep)

      INTEGER icell,nev,iev,irep
      REAL SY,SYi,GEr,GEri,beta
      INTEGER, DIMENSION(100) :: igauge
      REAL ds,vs,A,K,CP,S0,S,w0,fL,L0,Kv
      COMMON /CELULAS/ ds,vs,A,K,CP,S0,S,w0,fL,L0,Kv,igauge

      L0 = 10000*A/(2*w0)
      IF (S0.lt.0.090) THEN
         S = 10.8*SIN(ATAN(S0))+0.03
         ELSE
         S = 16.8*SIN(ATAN(S0))-0.50
      ENDIF
      beta = 11.16*SIN(ATAN(S0))/(3*(SIN(ATAN(S0))**0.8)+0.56)
      fL = (L0/22.1)**(beta/(beta+1))

      WRITE(*,400) ' Cell number...............................',icell
      WRITE(*,401) ' Area (ha)..................................',A
      WRITE(*,401) ' Soil density (-)..........................',ds
      WRITE(*,401) ' Sedimentation velocity (m/s)..............',vs
      WRITE(*,403) ' Drainage length w0 (m)....................',w0
      WRITE(*,403) ' Slope length L0 (m).......................',L0
      WRITE(*,401) ' Soil erodibility (ton.h/MJ/mm)............',K
      WRITE(*,402) ' Land-use factor CP (-)....................',CP
      WRITE(*,402) ' Average slope S0 (-) .....................',S0
      WRITE(*,401) ' Slope factor S (-)........................',S
      WRITE(*,401) ' Slope length factor L (-).................',fL
      WRITE(*,401) ' Vegetation parameter Kv ..................',Kv

      WRITE(21,400) ' Cell number ..............................',icell
      WRITE(21,401) ' Area (ha)..................................',A
      WRITE(21,401) ' Soil density (-)..........................',ds
      WRITE(21,401) ' Sedimentation velocity (m/s)..............',vs
      WRITE(21,403) ' Drainage length w0 (m)....................',w0
      WRITE(21,403) ' Slope length L0 (m).......................',L0
```

```fortran
      WRITE(21,401) ' Soil erodibility (ton.h/MJ/mm).............',K
      WRITE(21,402) ' Land-use factor CP ( - )....................',CP
      WRITE(21,402) ' Average slope S0 (-) .......................',S0
      WRITE(21,401) ' Slope factor S ( - )........................',S
      WRITE(21,401) ' Slope length factor L ( - )..................',fL
      WRITE(21,401) ' Vegetation parameter Kv ...................',Kv
      WRITE(21,400) ' Number of rainfall station ................',igauge(icell)
      IF (irep.eq.1) THEN
         WRITE(21,*)  '----------------------------------------------------------------------'
         WRITE(21,*)  ' id  gross-er(kg) Stream-pw(J/s/m2) Lambda(1/m) Lm(m) SDR SY(kg/ha) '
         WRITE(21,*)  '----------------------------------------------------------------------'
      ENDIF
      SY = 0
      GEr = 0
      iev = 0
      DO WHILE (iev.lt.nev)
         iev=iev+1
         CALL EVENT(icell,iev,GEri,SYi,irep)
         GEr = GEr + GEri
         SY = SY + SYi
      ENDDO
      WRITE(*,*) '------------------------------------------------------------------'
      WRITE(*,404) ' Total gross erosion (kg) in this cell ....',GEr
      WRITE(*,404) ' Total sediment yield (kg) ................',SY
      WRITE(*,405) ' Global sediment delivery ratio ...........',SY/GEr
      WRITE(*,*) '------------------------------------------------------------------'
      WRITE(21,*) '------------------------------------------------------------------'
      WRITE(21,404) ' Total gross erosion (kg) in this cell ....',GEr
      WRITE(21,404) ' Total sediment yield (kg) ................',SY
      WRITE(21,405) ' Global sediment delivery ratio ...........',SY/GEr
      WRITE(21,*) '------------------------------------------------------------------'

  400 FORMAT (a44,i6)
  401 FORMAT (a44,f9.3)
  402 FORMAT (a44,f9.4)
  403 FORMAT (a44,f9.2)
  404 FORMAT (a44,e10.4)
  405 FORMAT (a44,f5.3)
      END

!      5. SUBROUTINE THAT PROCESSES DATA FROM EACH EVENT

      SUBROUTINE EVENT(icell,iev,GEri,SYi,irep)

      INTEGER iev,irep,icell
      REAL Lm,SDR,eps,erosion,streamp,f2,L2
      REAL GEri,SYi
      INTEGER, DIMENSION(100) :: igauge
      REAL ds,vs,A,K,CP,S0,S,w0,fL,L0,Kv
```

```
      COMMON /CELULAS/ ds,vs,A,K,CP,S0,S,w0,fL,L0,Kv,igauge
      INTEGER, DIMENSION(10,3000) :: id
      REAL, DIMENSION(10,3000) :: D,dur,R
      COMMON /EVENTOS/ id,D,dur,R

!     id = event identity; D = runoff (mm/dur);
!     dur = event duration (min); R = erosivity (MJ.mm/ha/h)

      eps = R(igauge(icell),iev)*K*CP*S*fL/10
      erosion = 10000*A*eps
      GEri = erosion
      streamp = 9807*L0*S0*(D(igauge(icell),iev)/1000)/(60*dur(igauge(icell),iev))
      f2 = Kv*(ds/(ds-1))*streamp*L0/(9.807*eps*vs)

      CALL PARAM(L0,Lm,L2,SDR,f2)
      SYi = erosion*SDR
      IF (irep.eq.1) THEN
         WRITE(21,501) id(igauge(icell),iev),erosion,streamp,L2,Lm,SDR,SYi/A
      ENDIF
  501 FORMAT(i5,2x,e9.3,5x,e8.3,8x,e8.2,4x,f9.2,2x,f5.3,3x,f10.4)

      END

!     6. SUBROUTINE TO COMPUTE VARIABLE SDR AND PARAMETERS Lm & L2

    SUBROUTINE PARAM(L0,Lm,L2,SDR,f2)

    INTEGER i1
    LOGICAL run1
    REAL L0,Lm,L2,SDR,f2
    REAL Lm1,Lm2,Lm3,tol1,err1,nmax1
    REAL*8 h1,h2,h3,a,b,aux_log

    Lm1 = L0/100.
    x0 = L0-Lm1
    CALL Lambda(L0,f2,x0,Lm1,L2)
    a = L2*(Lm1+x0)
    b = L2*x0
    h1 = log(a - 1. - (b - 1.)*exp(-L2*Lm1)) - log(f2*L2) - log(1. - exp(-a))

    !print*,L2, f2, f2*L2, log(f2*L2)

    Lm2 = 50*L0
    x0 = 0.
    CALL Lambda(L0,f2,x0,Lm2,L2)
    a = L2*(Lm2+x0)
    b = L2*x0
    alfa = a/b
    h2 = log(a - 1. - (b - 1.)*exp(-L2*Lm2)) - log(f2*L2) - log(1. - exp(-a))
```

```
i1 = 0
tol1 = 0.001
nmax1 = 100.
run1 = .TRUE.
DO WHILE (run1)
    i1 = i1+1

    Lm3 = (ABS(h1)*Lm2+ABS(h2)*Lm1)/(ABS(h1)+ABS(h2))
    x0 = MAX(0.,L0-Lm3)
    CALL Lambda(L0,f2,x0,Lm3,L2)
    a = L2*(Lm3+x0)
    b = L2*x0

    aux_log = (b-1)*exp(-L2*Lm3)
    aux_log = (a-1) - aux_log
    aux_log = abs(aux_log)

    h3 = log(aux_log) - log(f2*L2) - log(1. - exp(-a))
    IF(h3*h2.le.0.) THEN
        Lm1 = Lm3
        h1 = h3
    ELSE
        IF(h3*h1.le.0.) THEN
            Lm2 = Lm3
            h2 = h3
        ELSE
            IF(ABS(h1).le.ABS(h2)) THEN
                Lm2 = Lm3
                h2 = h3
            ELSE
                Lm1 = Lm3
                h1 = h3
            ENDIF
        ENDIF
    ENDIF
    err1 = ABS(h3)
    IF (err1.le.tol1.or.i1.ge.nmax1) THEN
        run1 = .FALSE.
    ENDIF
ENDDO
Lm = Lm3
x0 = max(0.,L0-Lm)
CALL Lambda(L0,f2,x0,Lm,L2)

SDR = (L0-x0)/L0
SDR = SDR*(fexp(L2*Lm)-L2*(L0-x0)/2.)
SDR = SDR/fexp(L2*(x0+Lm))
```

```
END

!       7. SUBROUTINE TO COMPUTE PARAMETER LAMBDA-2, GIVEN L0, f2 AND Lm

SUBROUTINE Lambda(L0,f2,x0,Lm,L2)

INTEGER i2
LOGICAL run2
REAL L0,f2,x0,Lm,L2
REAL xm,L21,L22,L23,tol2,err2,nmax2
REAL*8 g1,g2,g3,c1, c2, c3

i2 = 0.

nmax2 = 100.
tol2 = 0.001
xm = (x0+L0)/2.

L21 = (5E-8)/Lm
c1 = L21*(0.5*(L0-x0) + Lm)
g1 = log(L21*Lm) + c1 - log(1. - exp(-L21*(x0+Lm)))

L22 = 0.01
c2 = L22*(0.5*(L0-x0) + Lm)
g2 = log(L22*Lm) + c2 - log(1. - exp(-L22*(x0+Lm)))

i2 = 0.
run2 = .TRUE.
DO WHILE (run2)
    i2 = i2+1.
    L23 = (ABS(g1)*L22+ABS(g2)*L21)/(ABS(g1)+ABS(g2))
    L23 = MAX(L23,(5E-8)/Lm)
    c3 = L23*(0.5*(L0-x0) + Lm)
    g3 = log(L23*Lm) + c3 - log(1. - exp(-L23*(x0+Lm)))

    IF(g3*g2.le.0.) THEN
        L21 = L23
        g1 = g3
    ELSE
        IF(g3*g1.le.0.) THEN
            L22 = L23
            g2 = g3
        ELSE
            IF(ABS(g1).le.ABS(g2)) THEN
                L22 = L23
                g2 = g3
            ELSE
                L21 = L23
```

```
                    g1 = g3
                ENDIF
            ENDIF
        ENDIF
        err2 = ABS(g3)
        IF (err2.le.tol2.or.i2.ge.nmax2) THEN
            run2 = .FALSE.
        ENDIF
    ENDDO
!       requirement due to numerical stability
    IF(L23*Lm.lt.5E-8) THEN
        L23 = (5E-8)/Lm
    ELSE
        IF (L23*Lm.gt.1.) THEN
            L23 = 1./Lm
        ENDIF
    ENDIF
        L2 = L23
END


!       Function that computes approximation of exp(x) - 1 using McLaurin series
      REAL FUNCTION fexp(x)
      REAL x
      fexp = x+(x**2)/2+(x**3)/6+(x**4)/24+(x**5)/120
      END
```

References

ANA: Rede Hidrometeorológica Nacional, URL https://metadados.ana.gov.br/geonetwork/, 2019.

Bagnold, R. A.: Bed load transport by natural rivers, Water Resources Research, 13, 303–312, https://doi.org/10.1029/wr013i002p00303, 1977.

de Araújo, J. C.: Entropy-based equation to assess hillslope sediment production, Earth Surface Processes and Landforms, 32, 2005–2018, https://doi.org/10.1002/esp.1502, 2007.

de Figueiredo, J. V., de Araújo, J. C., Medeiros, P. H. A., and Costa, A. C.: Runoff initiation in a preserved semiarid Caatinga small watershed, Northeastern Brazil, Hydrological Processes, 30, 2390–2400, https://doi.org/10.1002/hyp.10801, 2016.

Haan, C. T., Barfield, B. J., and Hayes, J. C.: Design hydrology and sedimentology for small catchments, Academic Press, Amsterdam, https://doi.org/10.1016/s0022-1694(96)90037-2, 1994.

Jaynes, E. T.: Information Theory and Statistical Mechanics, Physical Review, 106, 620–630, https://doi.org/10.1103/physrev.106.620, 1957a.

Jaynes, E. T.: Information Theory and Statistical Mechanics II, Physical Review, 108, 171–190, https://doi.org/10.1103/physrev.108.171, 1957b.

Mishra, S. K. and Singh, V. P.: Soil Conservation Service Curve Number (SCS-CN) Methodology, vol. 42, Springer, New York, https://doi.org/10.1007/978-94-017-0147-1, 2003.

Shannon, C. E.: A mathematical theory of communication, Bell system technical journal, 27, 379–423, https://doi.org/10.1002/j.1538-7305.1948.tb01338.x, 1948.

Snyder, F. F.: Synthetic unit-graphs, Eos, Transactions American Geophysical Union, 19, 447–454, 1938.

Soil Conservation Service: Soil Conservation Service National Engineering Handbook, section 4, Hydrology, Washington DC, 1972.

Wischmeier, W. H. and Smith, D. D.: Predicting rainfall erosion losses: a guide to conservation planning, Science and Education Administration, US Department of Agriculture, 1978.
